# Bio-ethanol production from wet coffee processing waste in Ethiopia

**DOI:** 10.1186/s40064-016-3600-8

**Published:** 2016-11-02

**Authors:** Asrat Gebremariam Woldesenbet, Belay Woldeyes, Bhagwan Singh Chandravanshi

**Affiliations:** 1 School of Chemical and Bio-Engineering, Addis Ababa Institute of Technology, Addis Ababa University, P.O. Box 385, Addis Ababa, Ethiopia; 2 Department of Chemistry, College of Natural Sciences, Addis Ababa University, P.O. Box 1176, Addis Ababa, Ethiopia

**Keywords:** Wet coffee waste, Hydrolysis, Fermentation, Bio-ethanol, Ethiopia

## Abstract

Large amounts of coffee residues are generated from coffee processing plants in Ethiopia. These residues are toxic and possess serious environmental problems following the direct discharge into the nearby water bodies which cause serious environmental and health problems. This study was aimed to quantify wet coffee processing waste and estimate its bio-ethanol production. The study showed that the wastes are potential environmental problems and cause water pollution due to high organic component and acidic nature. The waste was hydrolyzed by dilute H_2_SO_4_ (0.2, 0.4, 0.6, 0.8 and 1 M) and distilled water. Total sugar content of the sample was determined titrimetrically and refractometry. Maximum value (90%) was obtained from hydrolysis by 0.4 M H_2_SO_4_. Ethanol production was monitored by gas chromatography. The optimum yield of ethanol (78%) was obtained from the sample hydrolyzed by 0.4 M H_2_SO_4_ for 1 h at hydrolysis temperature of 100 °C and after fermentation for 24 h and initial pH of 4.5. Based on the data, it was concluded that reuse of the main coffee industry wastes is of significant importance from environmental and economical view points. In conclusion, this study has proposed to utilize the wet coffee processing waste to produce bio-ethanol which provides the alternative energy source from waste biomass and solves the environmental waste disposal as well as human health problem.

## Background

Presently economy of the world is mainly dependent on fossil energy sources like oil, coal, natural gas, etc., which are being used for the generation of fuel, electricity and other goods (Sarkar et al. 2012). Large consumption of fossil fuels has resulted in high levels of pollution (Demirbas and Demirbas 2011). Global energy consumption has increased gradually with the expansion of human population and increase of industrial prosperity. Use of transport fuel is affected by limited reserves of fossil fuel in the world (Sarkar et al. 2012).

Alternative energy sources must be technically feasible, economically competitive, environmentally acceptable, and readily available (Demirbas and Demirbas 2011). Several alternative fuels have been proposed, such as bio-ethanol, biodiesel, methanol, hydrogen, boron, natural gas,  and liquefied petroleum gas. Bio-ethanol is by far the most widely used bio-fuel for transportation worldwide (Balat 2011).

Africa is a large consumer of traditional sources of energy and faces energy insecurity for majority of its people. The availability and accessibility of socially and environmentally acceptable sources of energy are still very low and disproportionate between rural and urban areas. Except fuel wood, other energy sources (coal, crude oil and more recently bio-fuels) have been used as the major sources of power for the transport and industry sectors (van Zyl and Chimphango 2011).

Africa will have the largest potential for bio-energy production by 2050 in the world depending on the level of advancement of agricultural technology (van Zyl and Chimphango 2011). To realize this, advanced agricultural technologies and practices must be used in a sustainable way to meet the requirements of rural and urban communities, foster development of the industrial sector, reduce greenhouse gas emissions, develop agricultural infrastructure and lead to land restoration and ecologically healthy landscapes in Africa.

The current research efforts have become more focused on renewable, low-cost large-scale processes for lignocellulosic feedstocks originating mainly from agricultural and forest residues. Agricultural wastes do not have food value and do not demand separate land, water, and energy requirements (Sarkar et al. 2012; Limayem and Ricke 2012).

Lignocellulose is globally recognized as the preferred biomass for the production of a variety of fuels. It represents the most widespread and abundant source of carbon in nature to provide a sufficient amount of feedstock to satisfy the world’s energy needs in a renewable manner (van Zyl and Chimphango 2011). It has great potentials for the production of affordable fuel ethanol (Zheng and Pan 2009).

Lignocellulose materials are abundant renewable resource for the production of bio-fuel. The major components of lignocelluloses are cellulose, hemicellulose and lignin while the minor components are extractive liquid and ash. The major pathway for bio-ethanol production from biomass is biochemical conversion through saccharification and fermentation (Balat 2011).

Coffee by-products of wet processing constitute around 40% of the wet weight of the fresh fruit. Wet processing of coffee uses up to 15 m^3^ of water to produce one ton of clean beans (Kivaisi and Assefa 2010). Recently Woldesenbet et al. (2014, 2015a) have reported the characteristics of wet coffee processing waste and its management practice and environmental impact in Ethiopia. The wastes are disposed by dumping into the natural water systems or to nearby agricultural or grazing land in Ethiopia. Such ways of disposal have been the major health challenges to coffee farmers living in the surroundings of coffee processing plants.

Coffee processing waste contains large amounts of organic substrates which are suitable for bioconversion into value added bio-products (Kivaisi and Assefa 2010). Coffee pulp waste is generated in large quantities during wet coffee processing, which is known to contain 23–27% fermentable sugars on dry weight basis. Most of the coffee pulp waste remains underutilized in many countries (Adams and Ghaly 2007; Nayak and Harshitha 2012).

Therefore, large amount of unutilized biomass need to be changed into value added bio-products such as alternative energy sources and/or bio-ethanol which could also decrease environmental problems arising from waste disposal. Woldesenbet et al. (2015b) have made the theoretical estimation of bio-ethanol potential of wet coffee processing waste (pulp juice and mucilage). Hence, this study  was aimed to optimize the parameters (sulfuric acid concentration, hydrolysis temperature and time, and the fermentation time) for the production of bio-ethanol from wet coffee processing waste in Ethiopia and to characterize the sludge left after bio-ethanol production.

## Methods

### Sample collection

Mucilage and pulp juice (one barrel, 200 L each) were collected from Bonga, Teppi, Goma II and Limu Kosa wet coffee processing factories (Ethiopia). The samples were kept in ice box and transported to the Addis Ababa Institute of Technology, School of Chemical and Bio-Engineering and kept in the Environmental Engineering and Bio-Innovative laboratories.

### Chemicals

Ethyl alcohol (95%, Sigma-Aldrich, USA), sulfuric acid (98%, sd fine-chem. limited, Mumbai, India), sodium hydroxide (Abron Chemicals, India), hydrochloric acid (Abron Chemicals, India), methylene blue, Fehling solution (A and B), *Pichia anomala* (M4) (1% glucose, 0.5% peptone, 3% malt extract, and 2% agar–agar) and Whatman No. 1 filter papers were used in this research work.

### Apparatus and equipments

Porcelain crucibles (50 mL), Erlenmeyer flasks (250 mL), round bottom flasks (250 mL), pH meter (3505-JENWAY, UK), balance (HCB1002-ADAM, UK), stove (seven star, Germany), oven (202-OA, Germany), centrifuge (Thermo Fisher Scientific, USA), refractometry (Bellingham and Stanley, UK), gas chromatography (DANI, model GC-1000, Italy) and furnace (SX-2.5-12, box type resistance furnace, China) were used in this research work.

### Optimization of parameters

The procedure used for studying the optimization of all the parameters (including sterilization, hydrolysis time, temperature, acid concentration, fermentation time and temperature, distillation, and ethanol determination) is briefly described below. The effect of a particular parameter was studied by keeping all the parameters constant except the one under study.

To avoid microbial contamination, coffee waste was sterilized at 120 °C for 15 min, cooled to room temperature and kept in the fridge at 4 °C before use. The *P. anomala* (M4) was used for ethanol production optimization study. The yeast strain was maintained on agar slants (1% glucose, 0.5% peptone, 3% malt extract, and 2% agar–agar) was used to inoculate pre-fermenting media which contained 2% sugar and 3 g/L yeast extract and incubated for 12 h before use in the optimization studies (Viegas and Correia 1985). An aliquot of 100 mL of coffee waste were distributed into 250 mL conical flasks and pH was adjusted by using HCl and NaOH to 3.0, 3.5, 4.0, 4.5, 5.0, 5.5, 6.0 and 6.5. The flasks were sterilized at 120 °C for 15 min and upon cooling, 5 mL of the pre-fermentative yeast was inoculated to the sterilized media and there after incubated (without shaking) at an ambient temperature for 2 days. After every 12 h, 1.5 mL of the fermenting media was withdrawn to tubes. The tubes were centrifuged at 13,000 rpm for 4 min to get supernatant for ethanol analysis (Periyasamy and Venkatachalams 2009).

A series of experiments was performed for different hydrolysis times (30, 60, 90, and 120 min). In each experiment the hydrolysis time was kept constant as the fermentations times were varied from 12 to 48 h and the results were recorded. Suitable temperature for maximum production of ethanol was studied. Coffee waste was hydrolyzed with different concentrations of sulfuric acid (0.2, 0.4, 0.6, 0.8, 1 M and distilled water) in 500 mL Erlenmeyer flask and separately heated at 85, 100 and 115 °C for 1 h using oil thermostat. The hydrolysate obtained from hydrolysis of the coffee waste was collected and divided into four samples and used for the subsequent fermentation experiments. The fermentation was done at 12, 24, 36 and 48 h respectively withdrawing samples after 12 h, respectively (Dawson and Boopaty 2008). The ethanol produced was recovered and purified through distillation.

The ethanol produced from the fermentation process contains a significant quantity of water which must be removed. This was achieved by using the fractional distillation process by boiling the water and ethanol mixture. Since ethanol has a lower boiling point (78.3 °C) compared to that of water (100 °C), the ethanol turns into the vapor state before the water and it can be condensed and separated. The ethanol concentration in the distillate was determined gas chromatography (GC). Samples analyzed for ethanol production were taken at the end of fermentation after 12, 24, 36 and 48 h of incubation. Ethanol determination was done by using GC in which the sample was injected manually. Flame ionization detector was set at 280 °C. Separation was effected in a 30 m, 0.25 mm and 1 μm column (CP-SIL 8 CB) with the temperature maintained at 45–55 °C at a rate 2 °C/min for 5 min then at 10 °C/min to 200 °C. Column flow was employed at 1.5 mL/min with nitrogen as a carrier gas and hydrogen and compressed air as a combustion gases.

### Optimized procedure for ethanol production

The coffee waste was sterilized at 120 °C for 15 min, cooled to room temperature and kept in the fridge at 4 °C before use. The *P. anomala* (M4) was used for ethanol production. The yeast strain was maintained on agar slants (1% glucose, 0.5% peptone, 3% malt extract, and 2% agar–agar) was used to inoculate pre-fermenting media which contained 2% sugar and 3 g/L yeast extract and incubated for 12 h before use. An aliquot of 100 mL of coffee waste was transferred into a 250 mL conical flask and pH was adjusted to 4.5 using HCl and NaOH. The flask was sterilized at 120 °C for 15 min and upon cooling, 5 mL of the pre-fermentative yeast was inoculated to the sterilized media and there after incubated (without shaking) at an ambient temperature for 2 days. Coffee waste was hydrolyzed with 0.4 M  H_2_SO_4_ in 500 mL Erlenmeyer flask at 100 °C for 1 h using oil thermostat. The hydrolysate was  fermented for 24 h. The ethanol produced was recovered and purified through distillation. The ethanol concentration in the distillate was determined GC.

### Characterization of the sludge

The presence of nitrogen and trace elements in the fermentation media was determined according to the procedure described by Allen and Roche (1989). The minerals of the coffee waste were obtained by dry ashing method. About 5 g of coffee waste sludge was placed in a porcelain crucible and dried in an oven at 105 °C until a constant weight was obtained. The dried sludge was then placed in a muffle furnace and ignited at 550 °C for 2 h to get ash. The obtained ash was used for analysis of the minerals following the procedure described by Allen and Roche (1989).

### Statistical analysis

All the data were analyzed using SPSS 20 software. The results are presented as mean values of triplicate analysis.

## Results and discussion

### Initial pH optimization

**Fig. 1:**
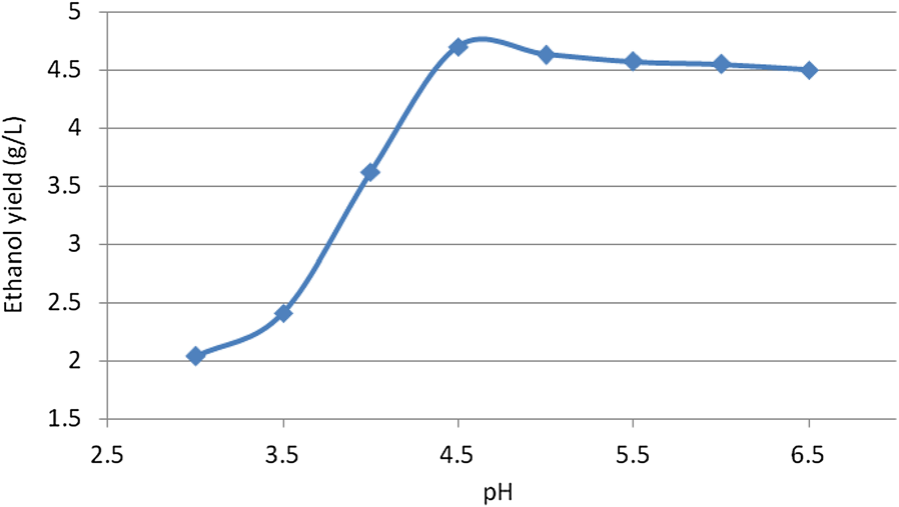
Effect of pH on ethanol production (the results were obtained on the hydrolysis of coffee waste at acid concentration of 0.4 M H_2_SO_4_, hydrolysis time of 1 h, and hydrolysis temperature of 100 °C, and fermentation time of 12 h)

Ethanol concentration increased with increase in pH to a maximum concentration of 4.7% (v/v) at pH 4.5, beyond which it started to show a slight decreasing trend. According to these results, pH 4.5 provides optimal condition for ethanol production. However, ethanol concentrations above pH 4.5 were almost equally high, suggesting that the natural pH of coffee waste (pH 5.5) can support yeast growth and ethanol production at appreciable levels. Thus coffee waste at its natural pH can be used in the production of bio-ethanol with little or no cost related to pH adjustments. Similar studies by Geetha and Kumar (2013) reported significant increase in ethanol yield from pH of 4.5–5.5, beyond which the levels did not increase much. The results are shown in the Fig. 1.

### Hydrolysis time

**Fig. 2:**
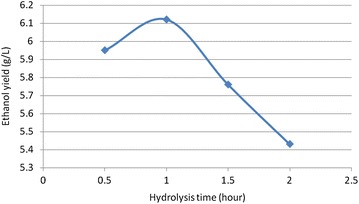
Effect of hydrolysis time on ethanol production (the results were obtained on the hydrolysis of coffee waste at the initial pH of 4.5, hydrolysis acid concentration of 0.4 M H_2_SO_4_ and hydrolysis temperature of 100 °C, and fermentation time of 24 h)

Prolonging the hydrolysis time significantly increased bio-ethanol concentration and then started to decline after 1 h hydrolysis time. The results are shown in Fig. 2.

Figure 2 shows that maximum bio-ethanol concentration of 6.12 g/L was achieved at 1 h hydrolysis time. However, as hydrolysis time increases above the optimum point, concentration of bio-ethanol decreases. This could have resulted because the longer residence (hydrolysis) time makes the sugars degraded to form inhibitors.

### Hydrolysis temperature

**Fig. 3:**
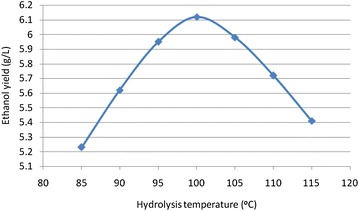
Effect of hydrolysis temperature on ethanol production (the results were obtained on the hydrolysis of coffee waste at the initial pH of 4.5, hydrolysis acid concentration of 0.4 M H_2_SO_4_ and hydrolysis time of 1 h, and fermentation time of 24 h)

It was observed that bio-ethanol concentration increases with increase in temperature and then started to decreases after 100 °C. The results were recorded and maximum yield was obtained at 100 °C. The results are shown Fig. 3.

Figure 3 shows that maximum bio-ethanol concentration of 6.12 g/L was achieved at 100 °C hydrolysis temperature. However, concentration of ethanol decreases after 100 °C.

### Fermentation time

**Fig. 4:**
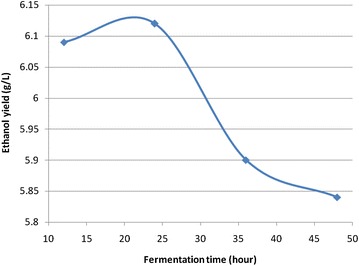
Effect of fermentation time on ethanol production (the results were obtained on the hydrolysis of coffee waste at initial pH of 4.5 at the optimized hydrolysis conditions (0.4 M H_2_SO_4_, 100 °C, 1 h)

This experiment was done based on optimized conditions of hydrolysis time and temperature. The fermentation was allowed for 2 days withdrawing samples after each 12 h interval. The results obtained are shown in Fig. 4.

The concentration of ethanol increased with increasing fermentation time and decreased at the end of fermentation time. Maximum ethanol concentration, 6.12 g/L was obtained after fermentation for 24 h. The decrease in concentration of bio-ethanol after 24 h might be due to the consumption of sugar by the microorganisms or the hydrolysate does contain significant levels of metabolic inhibitors that have accumulated and interferred the fermentation process.

### Bio-ethanol production from wet coffee processing waste under optimized conditions

Bio-ethanol production from wet coffee processing waste under different conditions was monitored by quantification of ethanol by GC. The gas chromatograms of bio-ethanol concentration at different conditions are summarized in Table 1.

**Table 1:** Summary of gas chromatogram of bio-ethanol concentration determination

Sample	Reten. time (min)	Area (mV s)	Height (mV)	Area (%)	Height (%)
Pure ethanol (95%)	0.639	5069	893	100	100
	Total	5069	893	100	100
Sample hydrolyzed by 0.2 M H_2_SO_4_	0.283	1623	241	31.3	23.7
	0.616	3565	774	68.7	76.3
	Total	5188	1014	100	100
Sample hydrolyzed by 0.2 M H_2_SO_4_ + 2 mL of pure ethanol (95%) added	0.276	2148	402	24.4	31.4
	0.639	6665	879	75.6	68.6
	Total	8813	1281	100	100
Sample hydrolyzed by 0.4 M H_2_SO_4_	0.286	2101	300	22.0	25.5
	0.632	7450	875	78.0	74.5
	Total	9551	1175	100	100
Sample hydrolyzed by 0.6 M H_2_SO_4_	0.283	2528	315	24.5	24.5
	0.609	7804	885	75.5	73.8
	Total	10,332	1199	100	100
Sample hydrolyzed by 0.8 M H_2_SO_4_	0.303	2624	183	29.6	21.0
	0.622	6233	688	70.4	79.0
	Total	8857	871	100	100
Sample hydrolyzed by 1 M H_2_SO_4_	0.280	3296	241	36.5	23.4
	0.616	5735	789	63.5	76.6
	Total	9031	1030	100	100
Sample hydrolyzed by distilled water	0.310	15,724	937	70.2	52.9
	0.636	6680	833	29.8	47.1
	Total	22,404	1770	100	100

Samples were hydrolyzed at 100 °C and fermented for 24 h

As it can be seen from Table 1 ethanol is detected by GC at the retention time of around 0.639 min and its area (%) for pure ethanol (95%) is 100%. For 0.2 M H_2_SO_4_ hydrolyzed sample, the peak is observed at relatively the same retention time with that of standard ethanol but the intensity of the peak for the sample is 68.7%. From which, it could be possible to say that the peak in the sample represents ethanol.

On addition of 2 mL of pure ethanol (95%) to the sample hydrolyzed by 0.2 M H_2_SO_4_, the peak intensity of the chromatogram of the sample increased from 68.7 to 75.6%. It can also be noticed that the retention time of the peak is almost similar but very small variation might be because the sample is injected manually. Therefore, the peak in the chromatogram observed around the retention time of 0.616 min for the sample hydrolyzed by 0.2 M H_2_SO_4_ and fermented for 24 h confirms the presence of ethanol in the sample.

The intensity of the peak for the sample hydrolyzed by 0.4 M H_2_SO_4_ is relatively higher than that of the sample hydrolyzed by 0.2 M H_2_SO_4_ which shows the concentration of ethanol increases with increase in concentration of H_2_SO_4_ used for hydrolysis up to the optimum point. The result still shows the presence of ethanol in 0.6, 0.8 and 1 M H_2_SO_4_ hydrolyzed samples but the intensity of the peak (concentration of ethanol) goes on decreasing after 0.4 M H_2_SO_4_. The concentration of ethanol is lowest in the sample hydrolyzed by distilled water compared to the samples hydrolyzed by different concentrations of H_2_SO_4_.

Table 2 presents the results of ethanol concentration produced from wet coffee waste hydrolyzed by different concentrations of sulfuric acid and distilled water and fermented at different times (12, 24, 36, 48 h) at optimized temperature and initial pH. It can be seen that maximum yield 78.0% is obtained from the sample hydrolyzed by 0.4 M H_2_SO_4_ after fermentation for 24 h.

**Table 2:** Ethanol concentration (%) obtained at different concentration of H_2_SO_4_ used for hydrolysis and at different fermentation time

Fermentation time (h)	0.2 M H_2_SO_4_	0.4 M H_2_SO_4_	0.6 M H_2_SO_4_	0.8 M H_2_SO_4_	1 M H_2_SO_4_	Distilled water
Ethanol concentration (%)						
12	50	58.5	56.3	54.2	45.8	29.7
24	68.7	78.0	75.5	70.4	63.5	29.8
36	67.1	74.4	67.4	64.4	63.5	27.8
48	53.1	71.3	65.3	61.7		

The results were obtained at optimized temperature, time and initial pH (100 °C, 1 h and pH of 4.5)

The comparison of bio-ethanol potential of coffee waste with different agricultural and agroindustrial wastes is given in Table 3.

**Table 3:** Bio ethanol production potential of different agricultural wastes

Feed stock	Ethanol production (g/L)	References
Sugarcane bagasses	10.2	Raghavendra and Havannavar (2007)
Banana peels	9.8	Manikandan and Saravanan (2008)
Poultry manure	5	Woldesenbet et al. (2013)
Coffee waste	6.12	This study

It is worthwhile to mention that the concentration of ethanol obtained (6.12 g/L) by the hydrolysis of the wet coffee waste is quite satisfactory compared to the maximum amount of ethanol obtained from the enzymatic fermentation of banana peels (9.8 g/L) and sugar bagasses (10.2 g/L).

The results obtained in this study show that coffee waste from wet coffee process has a potential for bio-ethanol production. Wet coffee waste has not been utilized in Ethiopia as a source of renewable energy. This has been the case despite the fact that Ethiopia is one of the most coffee growing countries.

Furthermore, coffee production residues can be analyzed under the concept of bio-refinery, in order to devise a more proper way of addressing the issue of adequate waste disposal and recovery, and environmental protection. The process of renewable energy from coffee waste can create a self-sustaining wet coffee milling process. Large scale wet coffee processing can produce large amounts of wet coffee waste. If the coffee waste is used in ethanol production it can result into sufficient amounts of ethanol. The ethanol can be blended up to 20% with petrol or diesel.

### Cost of bio-ethanol production

The bio-ethanol production cost from the Ethiopian wet coffee processing waste was assessed and it was estimated to be USD 0.45 L^−1^. Presently there is no bio-ethanol production investment in the Ethiopian wet coffee processing factories. The benefit cost ratio was also estimated to be >1.05, indicating that the investment in the production of bio-ethanol production from Ethiopian wet coffee processing waste is economically profitable. This study clearly indicates bio-ethanol production from the Ethiopian wet coffee processing waste is economically feasible.

### Characterization of the sludge

Coffee waste contained appreciable amount of minerals including nitrogen which are important in supporting growth of yeasts. As a result, no additional elements were used in the coffee waste during fermentation. The mineral content of coffee waste sludge is given in Table 4.

**Table 4:** Mineral content of coffee waste sludge

Mineral	Amount
Manganese	1.74 mg/kg
Magnesium	137 mg/kg
Zinc	5.28 mg/kg
Iron	45.5 mg/kg
Copper	4.02 mg/kg
Nitrogen	0.56%
Phosphorus	0.15%
Potassium	0.50%

Coffee waste is rich source of nutrients: 0.56% nitrogen; 0.15% phosphorus, and 0.5% potassium. It can be treated and used as organic fertilizer. Usually the coffee pulp is placed on piles and left to compost for about 3–4 months. During that time, it turns into black humus excellent for composting. Using organic fertilizers improves soil conditions and increases agricultural yield.

## Conclusion

Coffee waste is a good feedstock for bio-ethanol production when used in substantial quantities. The feasibility of ethanol production from coffee waste by means of dilute sulfuric acid hydrolysis was investigated. Distilled water and different concentrations of sulfuric acid were used for the hydrolysis of coffee waste to determine the most efficient concentration of acid and to ensure maximum ethanol yield. The optimization study showed that the highest ethanol concentration of 6.12 g/L was obtained under the optimum conditions of hydrolysis with 0.4 M sulfuric acid for 1 h by keeping the temperature at 100 °C, and fermentation time of 24 h with commercial baker’s yeast. This study has clearly showed the possibility of producing ethanol from wet coffee waste. The utilization of wet coffee waste as an alternative energy production reduces the environmental pollution and dependence on oil and petroleum in Ethiopia. It also provides alternative energy solutions for small-scale holders. From this study, it is thus possible to state that there is a potential for bio-ethanol production from wet coffee waste. A theoretical integrated model for joint production of biogas and bio-ethanol from wet coffee waste could also be formulated on a more sophisticated scale which is beyond the scope of this study.
